# Validation of an ultra-high performance liquid chromatography/UV method to quantify busulfan in plasma: application to therapeutic drug monitoring

**DOI:** 10.31744/einstein_journal/2025AO0964

**Published:** 2025-03-10

**Authors:** Laura Ben Olivo, Gabriel Giron Corrêa, Bruna Bernar Dias, Janaína Aparecida Risczik Arruda Corrêa, Bruna Martins Schweinberger, Raiza Lima do Carmo, Liane Esteves Daudt, Teresa Dalla Costa, Bibiana Verlindo de Araujo

**Affiliations:** 1 Pharmaceutical Sciences Graduate Program Faculdade de Farmácia Universidade Federal do Rio Grande do Sul Porto Alegre RS Brazil Pharmacokinetics and PK/PD Modeling Laboratory, Pharmaceutical Sciences Graduate Program, Faculdade de Farmácia, Universidade Federal do Rio Grande do Sul, Porto Alegre, RS, Brazil.; 2 Hematology and Flow Cytometry Unit Hospital de Clínicas de Porto Alegre Porto Alegre RS Brazil Hematology and Flow Cytometry Unit, Hospital de Clínicas de Porto Alegre, Porto Alegre, RS, Brazil.; 3 Graduate Program in Medicine: Medical Sciences Universidade Federal do Rio Grande do Sul Porto Alegre RS Brazil Graduate Program in Medicine: Medical Sciences, Universidade Federal do Rio Grande do Sul, Porto Alegre, RS, Brazil.; 4 Biochemistry Unit Hospital de Clínicas de Porto Alegre Porto Alegre RS Brazil Biochemistry Unit, Hospital de Clínicas de Porto Alegre, Porto Alegre, RS, Brazil.; 5 Hematology and Pediatric Bone Marrow Transplantation Service Hospital de Clínicas de Porto Alegre Porto Alegre RS Brazil Hematology and Pediatric Bone Marrow Transplantation Service, Hospital de Clínicas de Porto Alegre, Porto Alegre, RS, Brazil.

**Keywords:** Busulfan, Hematopoietic stem cell transplantation, Chromatography, high pressure liquid, Pharmacokinetics, Drug monitoring, Calibration, Hospital, public

## Abstract

**Objective:**

This study aimed to validate a sensitive, accurate, and precise bioanalytical ultra-high-performance liquid chromatography coupled with ultraviolet (UHPLC/UV) method for the determination of busulfan in human plasma using 1,6-bis-(methanesulfonyloxy) hexane as an internal standard for therapeutic drug monitoring.

**Methods:**

Plasma samples were deproteinized with acetonitrile (1:2, v/v) and, after derivatization with sodium diethyl dithiocarbamate, submitted to liquid-liquid extraction with ethyl acetate and evaporation at 50ºC under a nitrogen stream. Analyses were performed on a Shimadzu^®^ system using a C18 column and isocratic elution with methanol/water (70:30, v/v) at a flow rate of 0.4mL min^-1^ and detection at 277nm.

**Results:**

The retention times of busulfan and the IS were approximately 2.87 and 6.35 min, respectively. The plasma calibration curves were linear in the concentration range of 0.5-10 µg mL^-1^ with a coefficient of determination greater than 0.99. The lower limit of quantification was 0.5 µg mL^-1^. The inaccuracies and imprecisions of this method are less than 15%. The applicability of this method to pharmacokinetic studies was confirmed using patient samples obtained after 4 h of 3.2-5.4 mg kg^-1^ busulfan intermittent infusion.

**Conclusion:**

This method demonstrated the feasibility of quantifying samples within the target concentration range and quickly releasing results to allow for busulfan therapeutic monitoring.

## In Brief

Olivo et al. validated an in-house precise UHPLC/UV method for quantifying busulfan in human plasma for therapeutic monitoring. The method shows linearity (0.5–10 μg/mL) with a lower limit of quantification of 0.5 μg/mL, demonstrating accuracy and precision. It effectively supported therapeutic drug monitoring in a Brazilian public hospital by providing rapid and reliable results.

## Highlights

■ We validated the UHPLC/UV method for accurate busulfan quantification in plasma.

■ Inaccuracy and imprecision were below 15%, ensuring reliable therapeutic drug monitoring results.

■ This enables effective pharmacokinetic studies with rapid turnaround times in patient samples.

## Graphical abstract

**Figure:**
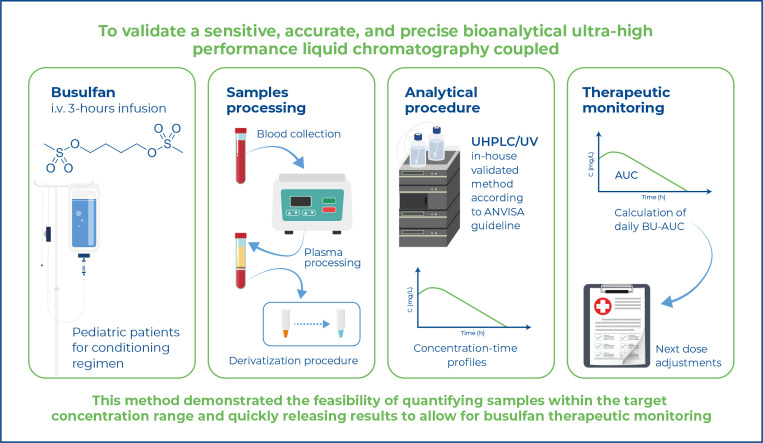

## INTRODUCTION

Busulfan (BU) (empirical formula: C6H14O6S2; molecular weight: 246,304g mol^-1^) is an alkylating agent used in conditioning regimens before hematopoietic stem cell transplantation (HSCT).^1,2^ Different conditioning protocols for HSCT are associated with BU and other antineoplastic drugs such as cyclophosphamide, fludarabine, thiotepa, melphalan, and gemcitabine. Busulfan conditioning regimen usually follows a 3-hour intravenous infusion, once or four times a day, for 4 d with doses varying from 3.2 to 5.1 mg kg^-1^per day.^1 3-6^

Similar to most alkylating agents, BU has a narrow therapeutic window. Its pharmacokinetics (PK) in children following 0.8 mg kg^-1^ intravenous infusion four times a day dose is described as one-compartment with linear elimination, peak plasma concentration (C_max_) of 3.6 µg mL^-1^ and time to C_max_ (t_max_) of 1.5-2.5 h, half-life (t_1/2_) of 2-3 h, volume of distribution (Vd) of 0.84 L kg^-1^ and clearance (CL) of approximately 0.29 L h^-1^kg^-1^.^7^ The therapeutic potential of BU is limited by its high PK variability, which directly affects clinical outcomes. High daily BU exposures (area under the curve, AUC_0-24_ >6000 µM L^-1^min^-1^) are associated with an increased risk of toxicity, particularly hepatic and neurological, while low exposures (AUC_0-24_ <3600 µM L^-1^min^-1^) are associated with HSCT failure.^8,9^

Maintaining a patient within the therapeutic range is crucial to obtain successful clinical outcomes after HSCT. However, maintaining patients within a therapeutic range is challenging.^8,9^ Therefore, BU therapeutic drug monitoring (TDM) is pivotal for ensuring HSCT success.

A validated bioanalytical method is necessary for TDM viewing to evaluate body exposure to BU. The method must provide fast, accurate, and precise results, allowing the evaluation of the AUC values from the first dose and the achievement of the target total body exposure on the four conditioning days.

Several methodologies have already been developed for BU quantification in plasma, including gas chromatography (GC) coupled with mass spectrometry (MS),^10-12^ GC coupled with electron capture,^13-15^ and high-performance liquid chromatography (HPLC) with MS.^16,17^ These are sensitive, specific, and effective methods, but they require expensive equipment and are thus difficult to use in the clinical routine of a public hospital. HPLC coupled with ultraviolet (UV) detection has become common to facilitate easy implementation in hospital routines.^18,19^ Although BU does not have a chromophore group in its chemical structure, it is possible to carry out a derivatization step, which has already been used for BU determination in human plasma samples, and its sensitivity is similar to that of GC methods.^20-22^

## OBJECTIVE

Considering the critical role of busulfan conditioning in hematopoietic stem cell transplantation, this study aimed to validate a fast bioanalytical method for busulfan quantification in human plasma using ultra-high-performance liquid chromatography coupled with UV (UHPLC/UV), considering the implementation of busulfan therapeutic drug monitoring in a Brazilian public hospital.

## METHODS

### Chemicals

Busulfan, sodium diethyl dithiocarbamate (DDTC), HPLC-grade ethyl acetate, acetonitrile, and methanol were obtained from Sigma-Aldrich (St. Louis, Missouri, USA). The internal standard (IS), 1,6-bis(methanesulfonyloxy) hexane, was synthesized by Toronto Research Chemicals. HPLC water from a Millipore Milli-Q system was used for the analysis.

### Preparation of standards and quality control samples

A standard stock solution of BU was prepared at a concentration of 1000 µg mL^-1^ in ethyl acetate, from which working solutions were prepared to obtain solutions with concentrations of 100 µg mL^-1^ and 10 µg mL^-1^. Appropriate dilutions were prepared to obtain working solutions at concentrations of 2.5, 3.75, 5.0, 12.5, 25.0, 37.5, and 50.0 µg mL^-1^. Likewise, quality control (QC) working solutions were made in four levels, with concentrations of 2.5 (lower limit), 7.5 (low), 20.0 (middle), and 40.0 µg mL^-1^ (high). The IS solution was prepared at a concentration of 100 µg mL^-1^ in ethyl acetate. A DDTC solution (8.2%) was prepared using ultrapure water.

For the calibration curves, the working solutions were diluted in plasma, as described below, resulting in standard samples with the following BU concentrations: 0.5, 0.75, 1.0, 2.5, 5.0, 7.5, and 10 µg mL^-1^. The QC samples had concentrations of 0.5 (lower limit of quantification, LLOQ), 1.5 (low, LQC), 4.0 (middle, MQC), and 8.0 µg mL^-1^ (high, HQC). The final concentration of the IS in all samples was 5 µg mL^-1^.

### Sample preparation

Blank human plasma was donated by the HCPA Blood Bank. Standard curve samples and quality control samples were prepared as follows: 160 µL of blank plasma was transferred to a polypropylene microtube. Then, 40 µL of BU working solution and 50 µL of IS solution were added. For plasma protein precipitation, 500µL of acetonitrile was used. After solvent addition, the mixture was vortexed for 60 s and centrifuged at 1,300 g for 5 min. Subsequently, 500 µL of the supernatant was transferred to a clean glass tube 200 µL of DDTC 8.2% were added and the mixture was vortexed for 10 s. Following, 2000 µL of ethyl acetate were added to the samples to extract the derivative, vortexed for 60 s and centrifuged at 1,300 g for 10 min. A 2000 µL aliquot of the organic phase was transferred to a clean glass tube and evaporated with nitrogen stream at 50^o^C. The dry extract was reconstituted with 100 µL of methanol, shaken in a vortex for 60 s, and transferred to vials of the UHPLC autosampler.

### Chromatographic conditions

Quantitative analysis of BU in human plasma were performed using an UHPLC LC-20AT (Shimadzu^®^, Kyoto, Japan), equipped with UV detector (SPD-M20A Shimadzu^®^, Kyoto, Japan), coupled to a SIL20A autosampler (Prominence, Kyoto, Japan), and to a CTO-20A column oven (Prominence, Kyoto, Japan), controlled by the Labsolutions software (Shimadzu^®^, Kyoto, Japan).

Chromatographic separation was performed using a C18 Kinetex^®^ reversed-phase column (50 × 2.1 mm), which was maintained at 40^o^C during the analysis. The isocratic mobile phase comprised methanol:water (70:30, *v/v*) at a flow rate of 0.4 mL min^-1^. The injection volume was 2 µL, the wavelength detection was set to 277 nm, and the run time was only 8 min.

### Method validation procedure

The validation of this bioanalytical method was conducted following the guidelines of the Brazilian Health Regulatory Agency (ANVISA - *Agência Nacional de Vigilância Sanitária*).^23^ The following tests were performed: calibration curve linearity, precision, accuracy, carryover effect, matrix effect, biological matrix specificity, and dilution tests.

The linearity of the method was established for a seven-point standard calibration curve using the area ratio (BU/IS) *versus* the nominal BU concentration. Analyses of blank plasma containing DDTC with and without IS were also conducted. Calibration curves were evaluated in triplicate (intra-day assay) on three different days (inter-day assay). The linearity was approved when the coefficient of variation was ≤20% of the nominal value for the LLOQ and ≤15% of the nominal value for the other calibration standards; at least 75% of the calibration standards in the curve were approved; and the coefficient of regression (R^2^) calculated as mean of 3 curves was >0.95.

The accuracy and precision were evaluated at four different levels of quality control (LLOQ, LQC, MQC, and HQC) and dilution QC (DQC) in five replicates (intra-day assay) on three different days (inter-day assay). The concentration of 15 µg mL^-1^ of the dilution control was obtained from a working solution of 75 µg mL^-1^. The accuracy was approved if the relative error was ± 20% for the LLOQ and ± 15% for other QC samples. The precision was approved if the coefficient of variation was ≤ 20% for the LLOQ and ≤ 15% for other control samples.

The carryover effect was determined by injecting a sequence of samples as follows: blank plasma sample, upper limit of quantification (10 μg mL^-1^) in duplicate, blank plasma sample in duplicate, and lower limit of quantification (0.50 μg mL^-1^). This test was considered approved if the interfering peak responses at the analyte retention time were less than 20% of the analyte response in the LLOQ samples and if the interfering peak responses at the IS retention time were less than 5% of the IS response.

The matrix effect was tested in nine samples with concentrations equivalent to the LQC and in nine samples with a concentration equivalent to that of the HQC. Each run included four normal, two lipemic, and two hemolyzed plasma samples from different sources and one sample in solution (methanol). The normalized matrix factor (NMF) was obtained using equation 1:


$$\mathrm{NMF}=\frac{\text{ analyte response in matrix / internal standard response in matrix }}{\text{ analyte response in solution / internal standard response in solution }}\times 100$$ Eq. 1

The method was considered approved if the coefficient of variation of the NMFs for all samples was less than 15%.

The specificity of the biological matrix was determined using six blank plasma samples (four normal, one lipemic, and one hemolyzed) and one LLOQ sample. The method was considered approved if the interfering peak responses at the analyte retention time were less than 20% of the analyte response in the processed LLOQ samples and if the interfering peak responses at the IS retention time were less than 5% of the IS response.

The stability of the spiked plasma samples was investigated in triplicate using LQC and HQC samples. Samples were analyzed right after preparation (time zero) and after storage at 15-25 ºC and 4-8^o^C for 18 h, 1 d, and 3 d. Samples were also stored at -20^o^C and analyzed after 18 h, 1 d, and 3 d. Two freezing/thawing (-20^o^C/room temperature) cycles were also evaluated. Post-processing stability was evaluated with processed samples kept in the auto-injector for 12 h. The stability was verified if the relative error was <15% when compared to the nominal value (time zero).

### Applicability of the validated method for therapeutic drug monitoring

The applicability of the UHPLC/UV method for TDM was tested in three pediatric patients (aged 4-9 years) who underwent HSCT and used BU as part of the conditioning regimen. The study was conducted after the first 3-hour intravenous infusion of BU 3.2 mg kg^-1^ BU, and the results were used to adjust the next doses for 4 d of conditioning viewing to reach the target exposition.

At specified intervals (240, 300, 360, and 420 min after the beginning of the infusion), blood samples were withdrawn via the central venous line into lithium heparin tubes. Blood samples were centrifuged (1,300 *g*, 10 min, at 4 ± 2^o^C) and the plasma was obtained and analyzed immediately after collection per the method developed. Daily AUC_0-∞_ was calculated according to plasma concentration through the trapezoidal method as shown by equations 2-4:


$$AUC_{0-t}=\left(\frac{c_{1}+c_{2}}{2}\right)\times \Delta t$$ Eq. 2

$$AUC_{ext}=\frac{C_{f}}{ke}$$ Eq. 3

$$AUC_{0-\infty }=\sum AUC_{0-t}+AUC_{ext}$$ Eq. 4

where AUC_0-t_ is the AUC up to the last blood sampling time, C represents the measured concentrations at different sampling times, Δt is the length of the sampling interval between two consecutive samples, AUC_ext_ represents the extrapolated area under the curve, C_f_ is the last measured concentration, ke is the elimination rate constant determined by the slope of the terminal phase of the concentration *vs*. time curve, and AUC_0-∞_ is the daily area under the curve. This work was approved by the Ethics Committee of the *Hospital de Clínicas de Porto Alegre* (CAAE: 69940317.7.0000.5347; # 2.713.246).

## RESULTS

**Figure 1:**
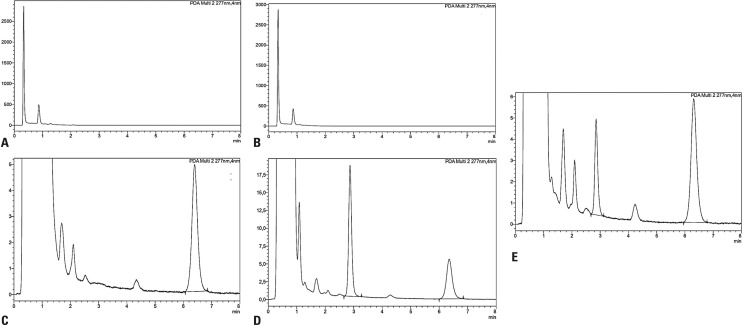
Representative chromatograms of busulfan in human plasma: (A) blank plasma; (B) blank plasma with DDTC; (C) plasma spiked with IS (5 µg/mL); (D) plasma spiked with busulfan and IS (10 and 5 µg/mL, respectively); (E) plasma from patient (20.2Kg) receiving 3.2 mg/kg of busulfan at 300 min sampling after the infusion dose (busulfan and IS 2.05 µg/mL and 5.5 µg/mL, respectively). Busulfan and IS retention times were observed at 2.87 min and 6.35 min, respectively

The method was selective, as can be observed in figure 1, where the chromatograms of BU and the IS are shown. No additional peaks due to endogenous substances were observed in the chromatogram beyond BU and the IS, indicating that the extraction method was selective for both molecules. The retention times of BU and the IS were approximately 2.87 min and 6.35 min, respectively.

The linearity of the method was observed through the calibration curve range (0.05-10μg mL^-1^), with a determination coefficient equal to or greater than 0.99 for all curves (Table 1). The LLOQ was 0.5µg mL^-1^, which was the lowest concentration with precision and accuracy per the ANVISA guidelines (≤20% for LLOQ) (Table 2).

**Table 1:** Busulfan bioanalytical method calibration curve parameters and statistics in human plasma

Validation day	Mean angular coefficient	Mean linear coefficient	Determination coefficient (R^2^)	Correlation coefficient (R)
1	0.989	-0.016	0.995-0.999	0.990-0.998
2	0.998	0.013	0.993-0.999	0.987-0.999
3	1.042	-0.062	0.997-0.999	0.994-0.998

**Table 2:** Intra- and inter-day precision and accuracy of the bioanalytical method to quantify busulfan in human plasma

Nominal concentrations	Mean (μg/mL)	SD	CV (%)	RE (%)
Intra-day variation				
0.5 μg/mL (LLOQ)				
1	0.60	0.03	5.3	10.4
2	0.58	0.05	7.8	17.8
3	0.57	0.04	4.7	14.8
1.5 μg/mL (LQC)				
1	1.50	0.12	8.1	-0.3
2	1.54	0.18	11.7	2.8
3	1.54	0.07	4.7	2.4
4.0 μg/mL (MQC)				
1	4.3	0.16	3.7	8.3
2	4.05	0.42	10.4	1.3
3	4.17	0.17	4.1	4.3
8.0 μg/mL (HQC)				
1	8.8	0.72	8.2	9.6
2	8.4	0.26	3.1	5.0
3	8.25	0.19	2.3	3.2
Inter-day variation				
0.5 μg/mL (LLOQ)	0.6	0.04	6.7	-12.5
1.5 μg/mL (LQC)	1.5	0.12	8.2	-1.6
4.0 μg/mL (MQC)	4.2	0.28	6.8	-4.4
8.0 μg/mL (HQC)	8.5	0.48	5.6	-5.6

Values (mean and SD.) represent n = 5/observations/day for intra- and 3 days for inter-day variation.

SD: standard deviation; CV: coefficient of variation; RE: relative error.

The precision and accuracy values are expressed as the coefficient of variation (CV%) and relative error (SE%), respectively, and the results are presented in table 2. The imprecision values for the intra-day and inter-day quality control samples were smaller than or equal to 11.7% and 8.2%, respectively. The intra-day and inter-day inaccuracy values for the quality control samples were less than or equal to 9.6% and 5.6%, respectively, proving that the method is precise and accurate according to the ANVISA acceptance criteria for bioanalytical methods.^23^ The DQC was diluted (1:1) to obtain the concentration for the analytical curve. The intra- and inter-day imprecision were smaller than or equal to 9.6% and 6.2%, respectively. Inaccuracy values for DQC were smaller than 4.4% for intra-day and equal to -3.8% for inter-day evaluation.

No carryover effects were observed in the BU or IS peaks, and the matrix effect test resulted in an NMF of 4.86%, which met the acceptance criteria (<15%).

Stability results are summarized in table 3. These studies showed that BU was stable under the conditions used in this study. The analyte was stable in plasma samples at room temperature up to 18 h and, under refrigerated (4-8 ^o^C) conditions, the samples remained stable for 3 d. After freezing at −20^o^C the stability was also guaranteed for 3 d. The analyte was stable after 2 cycles of freezing and thawing. Post-processing stability was maintained for 12 h in the autosampler.

**Table 3:** Busulfan plasma samples stability under different conditions

Condition	Stability duration	Low QC (Mean ± SD)	Low QC RE (%)	High QC (Mean ± SD)	High QC RE (%)
Room temperature (15-25 °C)	18 h	1.43±0.02	-5.10	7.49±0.18	-0.90
Refrigerated (4-8 °C)	3 d	1.53±0.11	1.30	7.25±0.13	4.10
Freeze (-20 °C)	3 d	1.48±0.05	-1.60	7.78±0.10	2.90
Freezing/Thawing (-20ºC/room temperature)	2 cycles	1.53±0.09	0.45	9.01±0.04	5.35
Post-Processing (autosampler)	12 h	1.34±0.02	-6.80	7.23±0.44	-8.70

RE: relative error; QC: quality control; Low QC nominal concentration: 1.5µg/mL; High QC nominal concentration: 8µg/mL.

**Figure 2:**
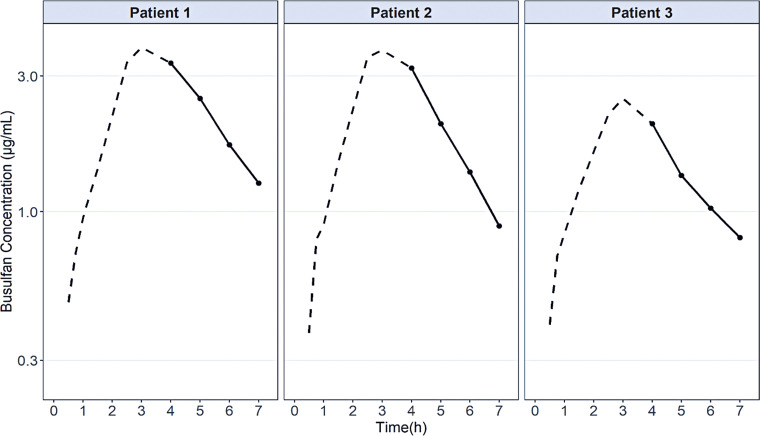
Concentration-time profiles of busulfan after the first 3-hour intravenous infusion of 3.2mg/kg in three pediatric patients undergoing conditioning regimen before hematopoietic stem cell transplantation

The adequacy of this method for the quantification of BU in patient samples was investigated. The individual concentration-time profiles of the three patients are shown in figure 2. As it can be seen, the elimination phase was adequately characterized, allowing the determination of the elimination rate constant, and the AUC_0-∞_. The mean AUC_0-∞_ was 3,371±787μM L^-1^min^-1^ (830±194mg min^-1^L^-1^) for the first dose, allowing the dose adjustment to guarantee adequate BU exposure between 14,400-24,000μM L^-1^min^-1^ by the end of the four conditioning days before HSCT. These results indicate that this analytical method is appropriate for measuring BU plasma concentrations, allowing BU dose individualization to guarantee the efficacious and safe use of this drug in pediatric patients.

## DISCUSSION

One of the problems related to BU quantification in biological samples is the lack of chromophore groups in the chemical structure. This makes it inadequate for UV detection without prior derivatization. Other detection methods, such as mass spectrometry, can overcome this problem. However, this type of equipment is not routinely available in most Brazilian public hospitals because this drug is currently being used. Because of the need for therapeutic monitoring due to its high toxicity, a fast and precise method with UV detection is fundamental to assuring patients undergoing conditioning regimens with adequate exposure to the drug before HSCT.

Derivatization of BU with DDTC, resulting in 1,4-bis (diethyldithiocarbamoyl) butane (DDCB), has been previously reported.^16-19^The resulting product is a molecule with intense light absorption that can be detected in the UV region. Given the need for a derivatization step, it is fundamental that the bioanalytical method uses an IS with similar chemical characteristics to be derivatized with BU and extracted together with DDCB, promoting greater control of the reaction efficiency. The IS used in this study was 1,6-bis(methanesulfonyloxy) hexane, which complied with both requirements. This IS has previously been used in other bioanalytical methods to quantify BU.^16-19^ The derivatization presented in this study was efficient and could be implemented routinely in hospitals, enabling TDM.

In summary, the bioanalytical method may improve TDM in clinical institutions for many reasons, such as time optimization, considering that the UHPLC/UV total running time of samples is only 8 min; low cost and simple sample preparation, as it does not require preliminary steps of sample filtration or the use of expensive extraction techniques; and accuracy and precision.

Two other studies were conducted in Brazil for the same purpose. Backes et al.^22^ and Effting et al.^24^ developed analytical methods for quantifying BU in human plasma by using HPLC with photodiode array (PDA) detection. Both methods used DDTC during sample preparation. However, some crucial differences were observed between their method and our method. First, the retention times described by Effting et al.^24^(17 min) and Backes et al.^22^ (8 min) were longer than those in our study (3 min). Secondly, the sample preparation described by Backes et al.^22^ (approximately 40 min) was too long compared to ours (approximately 15 min). Third, our volume of injection (2μL) is much lower than theirs (25 and 30μL), which is an important factor in analyzing pediatric samples due to the difficulty of getting plasma samples from children.^22,24^ Yet, our method was developed *in-house* using hospital equipment to quantify BU, which means that its application in clinical practice is practical. The proposed method can provide results in a short time and effectively assist with TDM and BU dose adjustments.

## CONCLUSION

The UHPLC/UV method validated in this study is fast, specific, precise, accurate, and reproducible for the quantification of busulfan in human plasma samples and can be used in routine laboratory examinations for drug therapeutic monitoring.
